# Priming increases the anti-tumor effect and therapeutic window of ^177^Lu-octreotate in nude mice bearing human small intestine neuroendocrine tumor GOT1

**DOI:** 10.1186/s13550-016-0247-y

**Published:** 2017-01-05

**Authors:** Johanna Dalmo, Johan Spetz, Mikael Montelius, Britta Langen, Yvonne Arvidsson, Henrik Johansson, Toshima Z. Parris, Khalil Helou, Bo Wängberg, Ola Nilsson, Maria Ljungberg, Eva Forssell-Aronsson

**Affiliations:** 1 Department of Radiation Physics, Institute of Clinical Sciences, Sahlgrenska Cancer Center, Sahlgrenska Academy, University of Gothenburg, SE-413 45 Gothenburg, Sweden; 2 Department of Pathology, Institute of Biomedicine, Sahlgrenska Cancer Center, Sahlgrenska Academy, University of Gothenburg, SE-413 45 Gothenburg, Sweden; 3 Department of Oncology, Institute of Clinical Sciences, Sahlgrenska Cancer Center, Sahlgrenska Academy, University of Gothenburg, SE-413 45 Gothenburg, Sweden; 4 Department of Surgery, Institute of Clinical Sciences, Sahlgrenska Cancer Center, Sahlgrenska Academy, University of Gothenburg, SE-413 45 Gothenburg, Sweden

**Keywords:** Neuroendocrine tumor, Xenograft model, Somatostatin receptors, ^177^Lu-DOTATATE, Fractionated radionuclide therapy, Gene expression, Radiation biology, MRI

## Abstract

**Background:**

^177^Lu-[DOTA^0^, Tyr^3^]-octreotate (^177^Lu-octreotate) is used for treatment of patients with somatostatin receptor (SSTR) expressing neuroendocrine tumors. However, complete tumor remission is rarely seen, and optimization of treatment protocols is needed. In vitro studies have shown that irradiation can up-regulate the expression of *SSTR1*, *2* and *5*, and increase ^177^Lu-octreotate uptake.

The aim of the present study was to examine the anti-tumor effect of a ^177^Lu-octreotate priming dose followed 24 h later by a second injection of ^177^Lu-octreotate compared to a single administration of ^177^Lu-octreotate, performed on the human small intestine neuroendocrine tumor cell line, GOT1, transplanted to nude mice.

**Results:**

Priming resulted in a 1.9 times higher mean absorbed dose to the tumor tissue per administered activity, together with a reduced mean absorbed dose for kidneys. Priming gave the best overall anti-tumor effects. Magnetic resonance imaging showed no statistically significant difference in tumor response between treatment with and without priming. Gene expression analysis demonstrated effects on cell cycle regulation. Biological processes associated with apoptotic cell death were highly affected in the biodistribution and dosimetry study, via differential regulation of, e.g., *APOE*, *BAX*, *CDKN1A*, and *GADD45A*.

**Conclusions:**

Priming had the best overall anti-tumor effects and also resulted in an increased therapeutic window. Results indicate that potential biomarkers for tumor regrowth may be found in the p53 or JNK signaling pathways. Priming administration is an interesting optimization strategy for ^177^Lu-octreotate therapy of neuroendocrine tumors, and further studies should be performed to determine the mechanisms responsible for the reported effects.

**Electronic supplementary material:**

The online version of this article (doi:10.1186/s13550-016-0247-y) contains supplementary material, which is available to authorized users.

## Background

^177^Lu-[DOTA^0^, Tyr^3^]-octreotate (^177^Lu-octreotate) is used to treat patients with somatostatin receptor (SSTR)-positive neuroendocrine (NE) tumors. The cure rate after ^177^Lu-octreotate is high in animal models xenotransplanted with human NE tumors [1–5]. In clinical studies, on the other hand, anti-tumor effects have been moderate, with complete remission in approximately 2% and partial remission in <30% of patients [6, 7]. Therefore, the clinical treatment protocol needs to be optimized. Optimization could be performed by either (1) reducing the absorbed dose to critical non-tumor tissues, and hence reduce detrimental side effects, in order to be able to increase the administered activity and thereby obtain better anti-tumor effects and/or (2) increase the therapeutic effects in tumor tissues without increasing the administered activity. The latter could be achieved by, e.g., up-regulation of SSTR expression in tumor cells, thereby increasing ^177^Lu-octreotate uptake in tumor tissue [8, 9].

We have previously demonstrated up-regulation of *SSTR* expression on NE tumor cells after ionizing radiation in vitro [10, 11]. Up-regulation of *SSTR1*, *2*, and *5* was found after irradiation, but there was no correlation to absorbed dose (0.12–8 Gy, X-rays). To clarify these mechanisms and obtain a better prediction of the effects in the clinical setting, appropriate animal models are needed. Human NE tumors xenografted to immunosuppressed mice provide such a model for evaluation of different treatment regimes. In a mouse xenograft model with the human small intestine-neuroendocrine tumor (SI-NET) GOT1 cell line xenotransplanted to nude mice, the uptake of a subsequent injection of 0.5 MBq ^111^In-octreotate in tumor was higher following an injection of 7.5 MBq ^177^Lu-octreotate (a non-curative amount) than following an injection of 30 MBq ^177^Lu-octreotate (a curative amount) [12, 13]. Furthermore, the optimal time between administration of ^177^Lu-octreotate and higher concentration of ^111^In-octreotide was 1 day or 3–13 days in GOT1 tumors on nude mice. The increased uptake of ^111^In-octreotate in tumor tissue may be due to up-regulation of SSTR expression. Based on these results, we hypothesize that a non-curative amount of ^177^Lu-octreotate as a priming dose increases the tumor uptake of subsequently administered ^177^Lu-octreotate in NE tumors, leading to an increased tumor-absorbed dose and anti-tumor effect.

Clinical studies frequently use fractionated therapy to better monitor toxicity effects. However, administered activities are often of similar size and separated by several weeks with the aim to reduce toxic effects rather than to increase the activity uptake in the tumor [14]. Furthermore, to our knowledge, there are no studies investigating the effects of a priming treatment schedule on either tumor or non-tumor tissues. Up-regulation of SSTR expression by a priming administration could potentially reduce the problem with receptor saturation in SSTR-expressing tumors when large amounts of ^177^Lu-octreotate are administered.

Before a new treatment protocol can be adopted, the effect of priming on uptake and absorbed dose to normal tissues should be evaluated. The critical organs in ^177^Lu-octreotate therapy are the bone marrow (acute effects, mostly transient) and the kidneys (late effects). The therapeutic window (or therapeutic index) gives the relation between the amount of ^177^Lu-octreotate that causes the therapeutic effect and the amount that causes toxicity.

The aim of the present study was to examine if a priming administration of ^177^Lu-octreotate 24 h before a subsequent ^177^Lu-octreotate administration increases the anti-tumor effect of ^177^Lu-octreotate in GOT1 tumor tissue in mice, compared with a single administration of the total amount of ^177^Lu-octreotate. Furthermore, we also determined the effect of priming on the biodistribution of ^177^Lu-octreotate to evaluate the effect on the therapeutic window.

## Methods

### Tumor model and animal handling

GOT1 (an SI-NET cell line) tissue was transplanted subcutaneously in the neck of 4-week-old female BALB/c nude mice (Charles River Laboratories International, Inc., Japan and Germany) as previously described [15]. Transplantation was performed under anesthesia using Ketaminol® vet. (Intervet AB, Sweden, 50 mg/ml) and Domitor® vet. (Orion Pharma Animal Health, Sweden, 1 mg/ml). Antisedan (Orion Pharma Animal Health, Sweden, 5 mg/ml) was used as an antidote after transplantation.

During the magnetic resonance imaging (MRI) experiments, the animals were anesthetized using a mixture of air and ~2% isoflurane (Isoba vet., Schering-Plough Animal Health, Farum, Denmark). Body temperature was maintained with a heating pad under the animal, and a pressure sensitive pad was used for respiratory triggering.

All ^177^Lu-octreotate or NaCl injections were administered i.v. into the tail vein. Water and autoclaved food were available ad libitum. At the end of the study, Pentobarbitalnatrium vet. (Apotek Produktion & Laboratorier AB, Sweden, 60 mg/ml) was administered i.p. followed by cardiac puncture.

### Radiopharmaceutical

^177^LuCl_3_ and [DOTA^0^, Tyr^3^]-octreotate were purchased from the Nuclear Research and Consultancy Group (IDB Holland, the Netherlands). Radiolabeling of [DOTA^0^, Tyr^3^]-octreotate with ^177^Lu was performed according to the manufacturer’s protocol. Quality control of the final ^177^Lu-octreotate solution was performed using instant thin layer chromatography (ITLC^TM^ SG, PALL Corporation, USA) with 0.1 M sodium citrate, pH 5 (VWR International AB, Sweden), as the mobile phase. The fraction of peptide-bound ^177^Lu was >98%, and the specific activity was approximately 26 MBq/μg octreotate. The ^177^Lu activity in each syringe was measured prior to and after administration of the radiopharmaceuticals with a well-type ionization chamber (CRC-15R, Capintec, USA). A Wallac 1480 gamma counter (WIZARD™ 3”, Wallac Oy, Finland) with a ±10% energy window over the 208-keV photon peak was used to measure the ^177^Lu activity in the samples.

### Overall study design

**Fig. 1:**
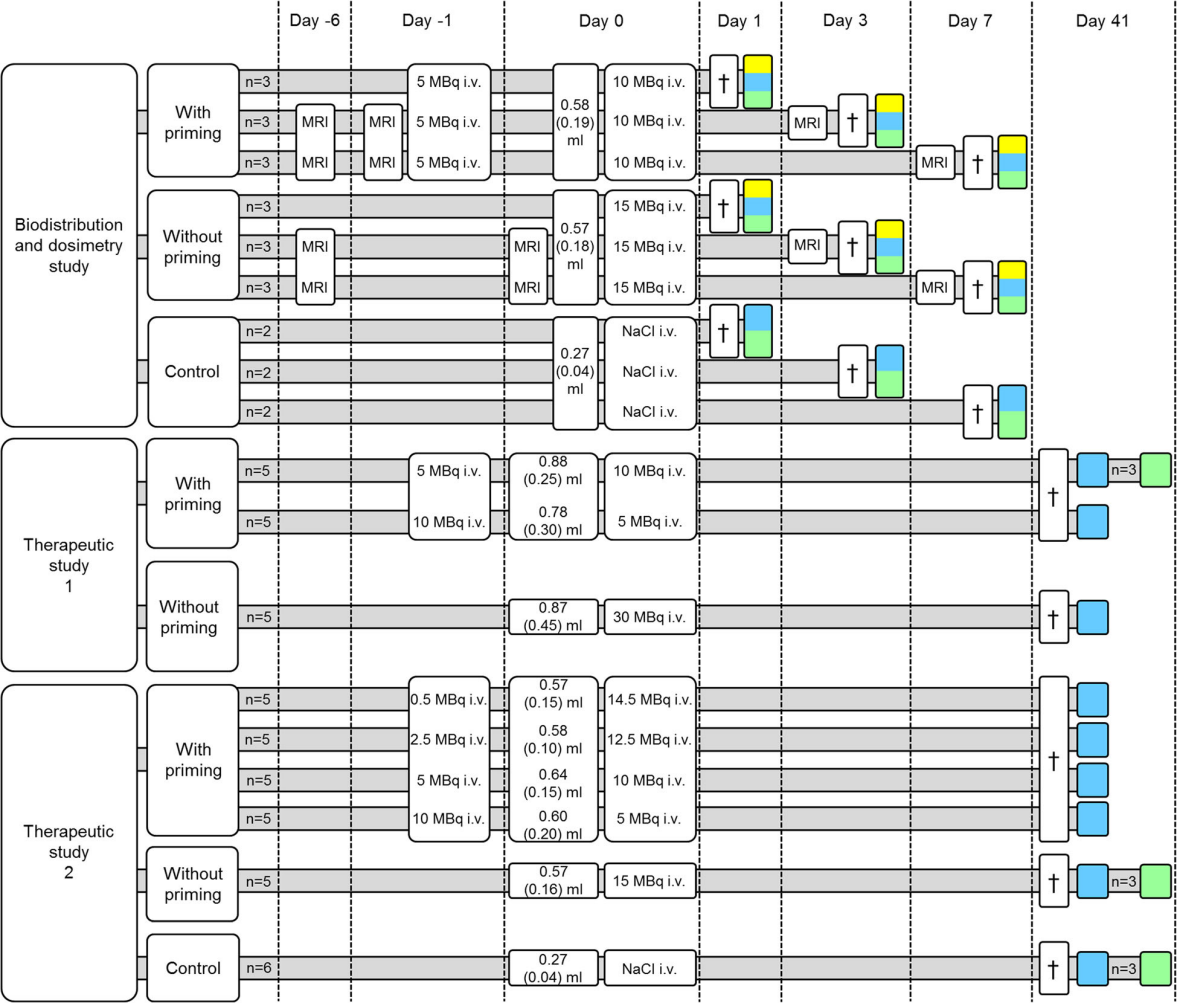
Experimental workflow. GOT1-bearing BALB/c nude mice were divided into three studies: one biodistribution and dosimetry study and two therapeutic studies. In these studies, tumor-bearing mice received either a single injection of ^177^Lu-octreotate or a priming administration followed by a subsequent injection of ^177^Lu-octreotate 24 h later. Magnetic resonance imaging (MRI), biokinetics, mean absorbed dose, tumor volume, tumor morphology, and gene expression of tumors were analyzed. *Dagger symbol* indicates that animals were killed and dissected; *yellow* indicates that radioactivity measurements and dosimetric calculations were performed on samples from adrenals, blood, kidneys, liver, lungs, pancreas, spleen, and tumor; *blue* indicates that tumor samples were fixed in formaldehyde, embedded in paraffin, and subjected to morphological and immunohistochemical analyses; *green* indicates that tumor samples were snap frozen in *l*N_2_ followed by RNA extraction and gene expression analysis

The present investigation consisted of three different studies: one biodistribution and dosimetry study including MRI examinations and two therapeutic studies. For an overview of experimental groups, see Fig. 1. We initially studied if different biodistributions and absorbed doses were obtained for tumor and normal tissues using two treatment schedules (biodistribution and dosimetry study). Then we compared the therapeutic effects of those schedules but also included other combinations of priming and subsequent injected amounts in order to study if other schedules gave similar effects or were more optimal (therapeutic studies).

In total, 70 GOT1 tumor-bearing nude mice (mean weight 21 g, SEM = 0.27 g) were included in the experiments. There was a large variation in tumor volume at the start of experiments (49–1400 mm^3^). Therefore, efforts were made to obtain a similar distribution of tumor size in all groups at the start of experiments (tumor volume distribution in the different groups is shown in Fig. 1). At the end of the experiments, tumor tissue from all animals were excised and divided into two halves: one half was instantly frozen in liquid nitrogen for gene expression analysis and the other half was weighed and placed in neutral buffered formaldehyde for radioactivity measurements and subsequent paraffin embedding.

### Biodistribution and dosimetry study

The biodistribution of ^177^Lu-octreotate in GOT1 tumor-bearing mice was determined as well as the effects on the activity concentration in tumor and normal tissues when a priming administration was given before a subsequent administration of ^177^Lu-octreotate.

Two groups (*n* = 9/group) of GOT1 tumor-bearing mice (5.1 months old) were given ^177^Lu-octreotate. One group received a priming administration of 5 MBq followed by 10 MBq of ^177^Lu-octreotate 24 h later. The other group received a single injection of 15 MBq ^177^Lu-octreotate. To avoid complete tumor remission in the evaluation of therapeutic effects after priming administration, a total activity of 15 MBq (0.6-μg peptide) was chosen. Animals were killed 24, 72, or 168 h after the last injection (*n* = 3/time point). Non-treated control animals (*n* = 2/time point) were injected with NaCl. Blood samples were collected by cardiac puncture. The adrenals, kidneys, liver, lungs, pancreas, spleen, and tumor were excised from each animal for ^177^Lu activity measurement using the gamma counter.

The ^177^Lu activity concentration in each tissue was calculated as percent of injected activity per gram tissue (%IA/g), and the tumor-to-normal tissue activity concentration ratio (T/N) was determined [16]. For animals receiving two injections (5 + 10 MBq) of ^177^Lu-octreotate, the ^177^Lu activity concentration was estimated for the 10 MBq administration only (in order to determine the biokinetics for the second administration, since the 5 MBq administration was supposed to act as priming administration and not as a therapeutic activity), based on measured ^177^Lu activity concentration (MBq/g) after 5 + 10 MBq and subtraction of the contribution from the priming administration. This contribution was estimated by assuming that the 5 MBq priming activity had the same biokinetics as the single administration of 15 MBq but extrapolated to the time points 48, 96, and 192 h after injection, corrected for radioactive decay.

The mean absorbed dose to the tissue was calculated according to the Medical Internal Radiation Dose Committee (MIRD) pamphlet 21 formalism [17]:

$$ D\left({r}_T,{T}_D\right)=\frac{\tilde{A}\left({r}_S,\ {T}_D\right){\displaystyle {\sum}_i}{E}_i{Y}_i\phi \left({r}_T\leftarrow {r}_S,\ {E}_i,{T}_D\right)}{M\left({r}_T,{T}_D\right)}, $$

where $\tilde{A}\left({r}_S,\ {T}_D\right)={\displaystyle {\int}_0^{T_D}A\left({r}_s,t\right)\ dt}$ is the time-integrated activity in the source organ *r*_*s*_ at the time of dose determination *T*_*D*_ = ∞. The mean energy emitted per nuclear transformation, ∑_*i*_E_*i*_Y_*i*_, was approximated for ^177^Lu to 147.9 keV/decay [18, 19], including β^−^ particles, Auger, and conversion electrons. The contribution from photons was not accounted for, and the target organ (*r*_*T*_) was set to be the same as the source organ (*r*_*s*_) in all calculations. This means that the mean absorbed dose to all organs might be somewhat higher (in the order of a few percent) than the data presented here. However, the absorbed fraction, *ϕ*(*r*_*T*_ ← *r*_*S*_, *E*_*i*_, *T*_*D*_), was set to 1 for all tumors and organs, which will give an overestimation in mean absorbed dose to the tumor of only up to approximately 10% [20]. *M*(*rT*,*T*_*D*_) was the mass of the tissue. The estimated absorbed doses are considered appropriate, especially when considering the uncertainties in pharmacokinetic data available.

### Therapeutic studies

Study 1: GOT1 tumor-bearing mice (11.5 months old) were injected with a priming administration of 5 or 10 MBq of ^177^Lu-octreotate followed by a second administration (24 h later) of 10 or 5 MBq ^177^Lu-octreotate, respectively. Thus, each mouse received a total activity of 15 MBq (0.6 μg peptide, *n* = 5/group), which was chosen to avoid complete tumor remission. A third group received 30 MBq (1.2 μg peptide) as a single injection (*n* = 5/group).

Study 2: GOT1 tumor-bearing mice (6.0 months old) were injected with a single administration of 15 MBq of ^177^Lu-octreotate, or different priming activities of 0.5, 2.5, 5, or 10 MBq of ^177^Lu-octreotate, followed by a second administration (24 h later) of 14.5, 12.5, 10, or 5 MBq ^177^Lu-octreotate, respectively, resulting in a total administrated activity of 15 MBq (0.6-μg peptide) to all groups (*n* = 5/group). Non-treated control animals (*n* = 6) were included in the study.

During the study period, tumor size and animal weight measurements were performed twice a week. Tumor size was determined using digital slide calipers by measuring the longest diameter *a* and the two perpendicular diameters *b* and *c*. Assuming the form of an ellipsoid, the tumor volume, *V*, was estimated

$$ V=\frac{\pi \cdot a\cdot b\cdot c}{6}. $$

The animals were killed 41 days after the last injection of ^177^Lu-octreotate. Tumor tissue was harvested and analyzed as described under the “Overall study design” section.

### MRI examinations

Animals from the biodistribution and dosimetry study (5 + 10 MBq: *n* = 6 and 15 MBq: *n* = 6) were each examined three times using MRI (Fig. 1): at day -6, day -1 (5 + 10 MBq group), day 0 (15 MBq group), day 3 (*n* = 3 mice/group), and day 7 (*n* = 3 mice/group). Tumor volume was determined using a caliper prior to all MRI examinations, and the animals were killed after the third examination. All tumors were resected and weighed for volume determination (assuming a density of 1 g/cm^3^).

MRI experiments were performed using a dedicated small animal 7 T MRI system (Bruker BioSpin MRI GmbH, Germany; software: ParaVision 5.0) equipped with a 72-mm transmit volume coil and a 4-channel array rat brain receiver coil (RAPID Biomedical GmbH, Germany). A T2-weighted, 2D RARE (rapid acquisition with relaxation enhancement) sequence was used with respiration triggering and fat suppression (repetition time, 4200 ms; effective echo time, 30 ms; signal averages, 3; turbo factor, 4) [21]. Image field-of-view covered the tumor, using ~160 * 160 μm^2^ pixel size and 700-μm slice thickness (contiguous slices).

**Fig. 2:**
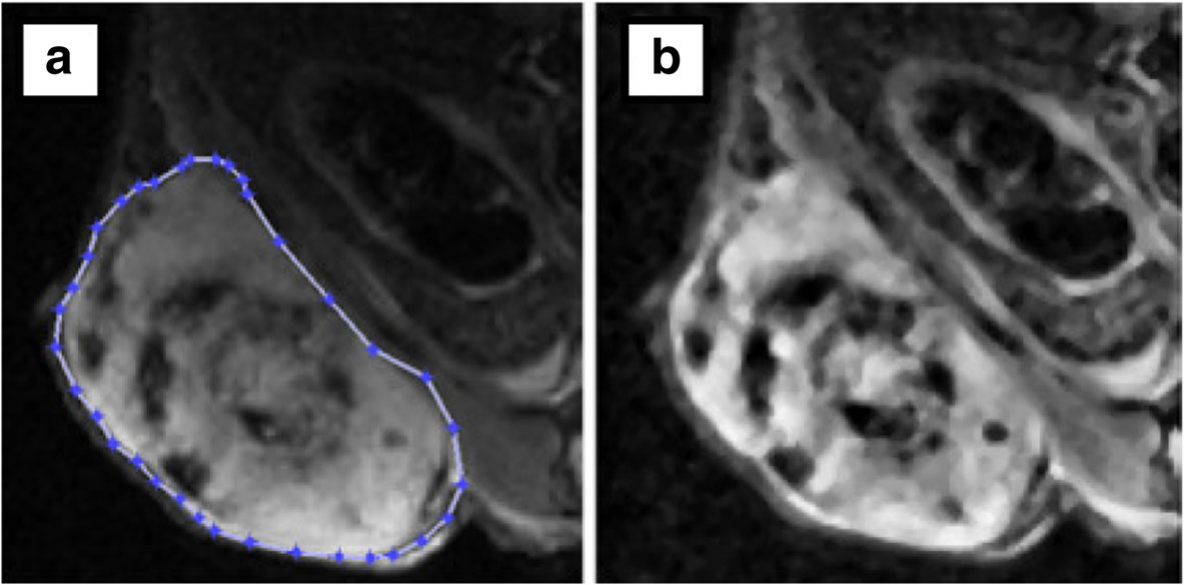
MRI of a GOT1 xenograft. **a** MRI of a representative GOT1 tumor (animal no. 3 in the 5 + 10 MBq group imaged on day -1, prior to priming administration) after delineation for volume calculation. The fluctuating intra-tumor intensity illustrates the heterogeneity of tumor tissue, containing both viable (hyper-intense) and necrotic (hypo-intense) regions. The coil sensitivity profile makes tissues closer to the coil (*bottom of image*) brighter than more distant tissues, which confounds histogram analysis. This is corrected for in **b** where histogram equalization (correcting for coil sensitivity profile) and 3 × 3 median noise filtering has been applied

Images were processed on a PC workstation using an in-house-developed MATLAB software (R2011b, The MathWorks, USA). All images were subjected to histogram equalization for homogeneity correction (due to the surface coil sensitivity profile) and a 3 * 3 median filter to reduce random noise (Fig. 2a, b). Each tumor was manually delineated on all slices, and volume was calculated by multiplying the total number of voxels with the voxel volume [22]. Tumor volumes determined by MRI and calipers at the day of sacrifice were compared with the volumes of the resected tumors. Histograms were created of the voxel intensity distribution within each tumor, and the histogram skewness was used as a surrogate marker of tumor viability, where a positive skewness would indicate an abundance of low-intensity voxels, typical of necrotic regions, and a negative skewness would indicate a greater amount of viable tumor tissue, typically seen as high-intensity regions in T2-weighted images.

### Transcriptional analysis

Frozen tumor tissue was homogenized using the TissueLyser LT (Qiagen, Germany), and total RNA was extracted using the RNeasy Lipid Tissue Mini Kit (Qiagen) according to the manufacturer’s instructions. RNA quantity was determined using an ND-1000 Spectrophotometer (NanoDrop Technologies, DE, USA), and RNA quality was determined with the RNA 6000 Nano LabChip Kit and Agilent 2100 Bioanalyzer (Agilent Technologies, CA, USA). RNA samples with RNA integrity numbers above 6.0 were used for gene expression analysis.

Gene expression analysis was performed on tumor tissues from three animals per time point from the 5 + 10 MBq and 15 MBq groups at 24 h, 72 h, 168 h, and 41 days, as well as control animals, injected with NaCl solution for each time point (*n* = 2 at 24, 72, and 168 h; *n* = 3 at 41 days, Fig. 1). Hybridization of the RNA samples was performed at the Swegene Center for Integrative Biology (SCIBLU, Lund University, Sweden) on Illumina HumanHT-12 v4 Whole-Genome Expression BeadChips (Illumina, CA, USA), containing 47,231 probes per array. The BeadChips were analyzed using the Illumina iScan N240 microarray scanner (Illumina, CA, USA). Data pre-processing and quantile normalization were performed on the raw signal intensities using the BioArray Software Environment (BASE) system. Further processing was then performed using Nexus Expression 3.0 (BioDiscovery, CA, USA) as previously described [23, 24]. The Gene Ontology (GO) database (http://www.geneontology.org) was used for the analysis of associated biological processes [23]. Emphasis in this study was placed on the analysis of differential regulation associated with cell death and cell cycle regulation, as these factors have previously been described as major actors in tumor radiation response in the clinical setting [25, 26]. The gene expression data in this study have been deposited in NCBI’s Gene Expression Omnibus (GEO accession GSE80022).

The expression levels of *SSTR1–5* in the biodistribution and dosimetry study were analyzed using quantitative reverse transcription-polymerase chain reaction (qRT-PCR) with predesigned TaqMan® assays (Applied Biosystems, CA, USA) specific for *SSTR1–5* (Hs00265617_s1 (*SSTR1*), Hs00990356_m1 (*SSTR2*), Hs01066399_m1 (*SSTR3*), Hs01566620_s1 (*SSTR4*), Hs00990407_s1 (*SSTR5*)), *GAPDH* (Hs99999905_ml), and *TUBB* (Hs00742828_s1). The PCR reactions were performed in triplicate using a 7500 Fast Real-Time PCR System (Applied Biosystems). The levels of mRNA expression are reported relative to the two housekeeping genes *GAPDH* and *TUBB* (2^−(Ct(target gene) − Ct((*GAPDH* + *TUBB*)/2))^). All qRT-PCR reactions were performed on complementary DNA (cDNA), synthesized from the same RNA extracted for use in the microarray experiments, using SuperScript™ III First-Strand Synthesis SuperMix (Invitrogen, CA, USA). cDNA reactions without addition of reverse transcriptase prior to qRT-PCR did not monitor any interfering genomic DNA.

### Morphology and immunohistochemistry

At the end of the experiments, all xenografts were harvested and processed for microscopic examination. The morphology of formalin-fixed and paraffin-embedded xenografts was examined after hematoxylin-eosin and Masson’s trichrome staining. For immunohistochemical analysis, sections were placed on positively charged glass slides and treated with EnVision™ FLEX Target Retrieval Solution (high pH) using a PT-Link (Dako, Denmark). Immunohistochemical staining was performed in an Autostainer Link using EnVision™ FLEX according to the manufacturer’s instructions (Dako). Positive and negative controls were included in each run. Primary antibodies to chromogranin A (MAB319; Merck Millipore, Germany), synaptophysin (Sy38, Dako), Ki67 (AB9260; Merck Millipore), and BAX (B-9; sc-7480; Santa Cruz Biotechnology, CA) were used.

The effects of ^177^Lu-octreotate on xenografted tumors were evaluated morphologically and graded according to Becket et al. [27]. The percentage of Ki67-positive tumor cell nuclei was calculated by manual counting of printed images from “hot spots,” including >500 tumor cell nuclei. The intensity of BAX staining was evaluated by two independent observers and graded as absent, weak, moderate, or strong.

### Statistical analyses

All tumor volume measurements and results from the biodistribution data for each group were expressed as mean value and standard error of the mean (SEM) or standard deviation (SD). Student’s *t* test was used to analyze data throughout all studies between groups and within groups at different time points using a two-tailed unpaired *t* test, and *p* < 0.05 was considered statistically significant.

The difference between groups regarding skewness analyses of the MRI data after treatment was assessed using an *F* test, where the skewness vs. time regression coefficients was compared. In this test, the interaction of time and treatment was considered.

The differential expression of transcripts (treated vs. untreated) was determined from unfiltered microarray probes using the Benjamini-Hochberg method with adjusted *p* < 0.01 [28]. Transcripts with ≥1.5-fold change were considered significantly regulated compared to controls. Enrichment analysis on gene sets to identify over-represented GO terms for biological processes was performed using Fisher’s exact test with *p* < 0.05 considered statistically significant.

## Results

### Biodistribution and dosimetry of ^177^Lu-octreotate is significantly affected by priming administration

Mean ^177^Lu activity concentration in GOT1-bearing mice at 24, 72, and 168 h after the last injection is presented in Table 1 (A). Compared with the values at 24 h, the mean activity concentration in the tumor increased 72 h after priming but decreased for the single administration (15 MBq), with a statistically significant difference between the treatment schedules at 72 h. In the kidneys, the activity concentration decreased equally in both groups, as was observed for the blood. One tumor from the 15 MBq group (killed 72 h after injection) showed only background levels of ^177^Lu activity concentration. This tumor did not show the typical hyper-intensity in the MRI examinations which was observed for all other tumors in the study. The RNA extraction process also failed to produce sufficient RNA quantities for analysis from this tumor sample, and histological examination showed no viable tumor cells. The tumor was therefore excluded from all analyses, but non-tumoral tissues from this animal were still included in the activity concentration and dosimetry calculations.

**Table 1:** Biodistribution of ^177^Lu-octreotate in GOT1-bearing nude mice

A	^177^Lu activity concentration (%IA/g)								
	With priming, (5+) 10 MBq (*n* = 9)			Without priming, 15 MBq (*n* = 8)					
Tissue	24 h	72 h	168 h	24 h	72 h	168 h			
Adrenals	1.4 (0.1)^a^	0.42 (0.20)^a^	0.36 (0.06)	0.60 (0.03)^a^	1.4 (0.1)^a^	0.30 (0.06)			
Blood	0.013 (0.001)	0.0048 (0.0005)	0.0017 (0.0004)	0.011 (0.001)	0.0055 (0.0002)	0.0021 (0.0002)			
Kidneys	4.5 (0.8)	1.5 (0.1)	0.71 (0.14)^a^	6.8 (0.3)	2.3 (0.4)	0.50 (0.04)^a^			
Liver	0.094 (0.008)	0.077 (0.009)	0.070 (0.009)	0.12 (0.06)	0.10 (0.01)	0.049 (0.004)			
Lungs	0.84 (0.10)^a^	0.43 (0.03)	0.31 (0.02)^a^	0.48 (0.04)^a^	0.33 (0.02)	0.15 (0.003)^a^			
Pancreas	0.54 (0.02)^a^	0.30 (0.02)	0.18 (0.02)^a^	0.36 (0.03)^a^	0.26 (0.04)	0.062 (0.011)^a^			
Spleen	0.080 (0.010)	0.090 (0.006)	0.090 (0.010)	0.10 (0.01)	0.088 (0.009)	0.061 (0.005)			
Tumor	2.5 (0.9)	4.6 (0.8)^a^	1.8 (0.5)	1.6 (0.1)	1.6 (0.2)^a,b^	0.84 (0.31)			
B	Tumor-to-normal tissue activity concentration ratio (T/N)								
	With priming, (5+) 10 MBq (*n* = 9)			Without priming, 15 MBq (*n* = 8)					
Tissue	24 h	72 h	168 h	24 h	72 h	168 h			
Adrenals	1.7 (0.5)	23 (9)	4.6 (1.2)	2.7 (0.4)	1.1 (0.1)	3.8 (1.6)			
Blood	190 (62)	1100 (280)	1100 (360)	160 (34)	290 (21)	420 (160)			
Kidneys	0.62 (0.29)	3.1 (0.7)^a^	2.6 (0.8)	0.23 (0.02)	0.76 (0.19)^a^	1.5 (0.6)			
Liver	27 (10)	63 (14)	24 (5)	14 (1)	17 (2)	16 (6)			
Lungs	2.9 (1.1)	10 (2)	5.4 (1.3)	3.4 (0.4)	4.9 (0.6)	5.8 (2.2)			
Pancreas	4.5 (1.5)	15 (2)^a^	10 (3)	4.4 (0.3)	6.6 (1.1)^a^	12 (4)			
Spleen	32 (13)	53 (11)	18 (4)	17 (1)	19 (3)	13 (5)			
C	Tumor cell proliferation assed by Ki67 staining (%)								
	With priming, (5+) 10 MBq (*n* = 9)			Without priming, 15 MBq (*n* = 8)			Controls		
Antibody	24 h	72 h	168 h	24 h	72 h	168 h	24 h	72 h	168 h
Ki67	27 (4)	22 (2)^c^	25 (2)^c^	31 (1)^c^	29 (3)^b^	28 (2)^c^	45 (1)^b^	42 (1)^b^	42 (2)^b^

A: ^177^Lu activity concentration (%IA/g), B: tumor-to-normal tissue activity concentration ratio, and C: percent of tumor cells with positive immunohistochemical staining from tumors after 5 + 10 or 15 MBq ^177^Lu-octreotate with or without priming activity, respectively, corrected for physical decay, in different tissues in GOT1 tumor-bearing nude mice at 24, 72, and 168 h after injection (*n* = 3/group). Values for the 5 + 10 MBq group (activity concentration and T/N) were estimated based on data from the 10 MBq administrations only i.e. therapeutic effect. Values are given as mean (SEM). Statistically significant difference (*p* < 0.05) between priming and single administration and between treated animals and controls is indicated by ^a^ and ^c^, respectively, ^b^ *n* = 2

For all normal tissues except the spleen (with priming) and adrenals (without priming), the activity concentration decreased with time. The activity concentration after administration without priming had an initial higher uptake in the kidneys, liver, and spleen, but the activity concentration decreased faster than in the priming group to the last time point measured. In the pancreas and lungs, the activity concentration decreased with time for both groups but was higher when using priming.

The mean T/N ratios are presented in Table 1 (B). The T/N ratios for the blood, kidneys, liver, and spleen were higher when a priming administration was given. The pancreas, adrenals, and lungs in the priming group had a peak in T/N ratio after 72 h and decreased thereafter, while the T/N ratio increased with time in the group without priming. There was a statistically significant difference in T/N ratios in the kidneys and pancreas at 72 h between animals receiving treatment with and without priming.

The estimated mean absorbed doses with or without priming are given in Table 2. The mean absorbed dose to the tumor per administered activity was 0.51 Gy/MBq after priming and 0.27 Gy/MBq without priming administrations. The kidneys received a lower mean absorbed dose per administered activity with priming compared to no priming (0.25 vs. 0.32 Gy/MBq). Priming resulted in higher mean absorbed dose per administered activity for the adrenals, lungs, pancreas, and spleen.

**Table 2:** Mean absorbed dose calculations of ^177^Lu-octreotate in GOT1-bearing nude mice

Tissue	Mean absorbed dose per administered activity (Gy/MBq)	
	With priming, (5+) 10 MBq	Without priming, 15 MBq
Adrenals	0.11	0.079
Blood	0.00067	0.00069
Kidneys	0.25	0.32
Liver	0.014	0.011
Lungs	0.069	0.038
Pancreas	0.042	0.024
Spleen	0.023	0.012
Tumor	0.51	0.27^a^

The mean absorbed dose per administered activity after 10 or 15 MBq ^177^Lu-octreotate (Gy/MBq) (with and without priming, *n* = 9/group), respectively. Values for the 5 + 10 MBq group were estimated from the values for the 10 MBq administrations only

^a^ *n* = 8

### Increased anti-tumor effect of ^177^Lu-octreotate after priming administration

**Fig. 3:**
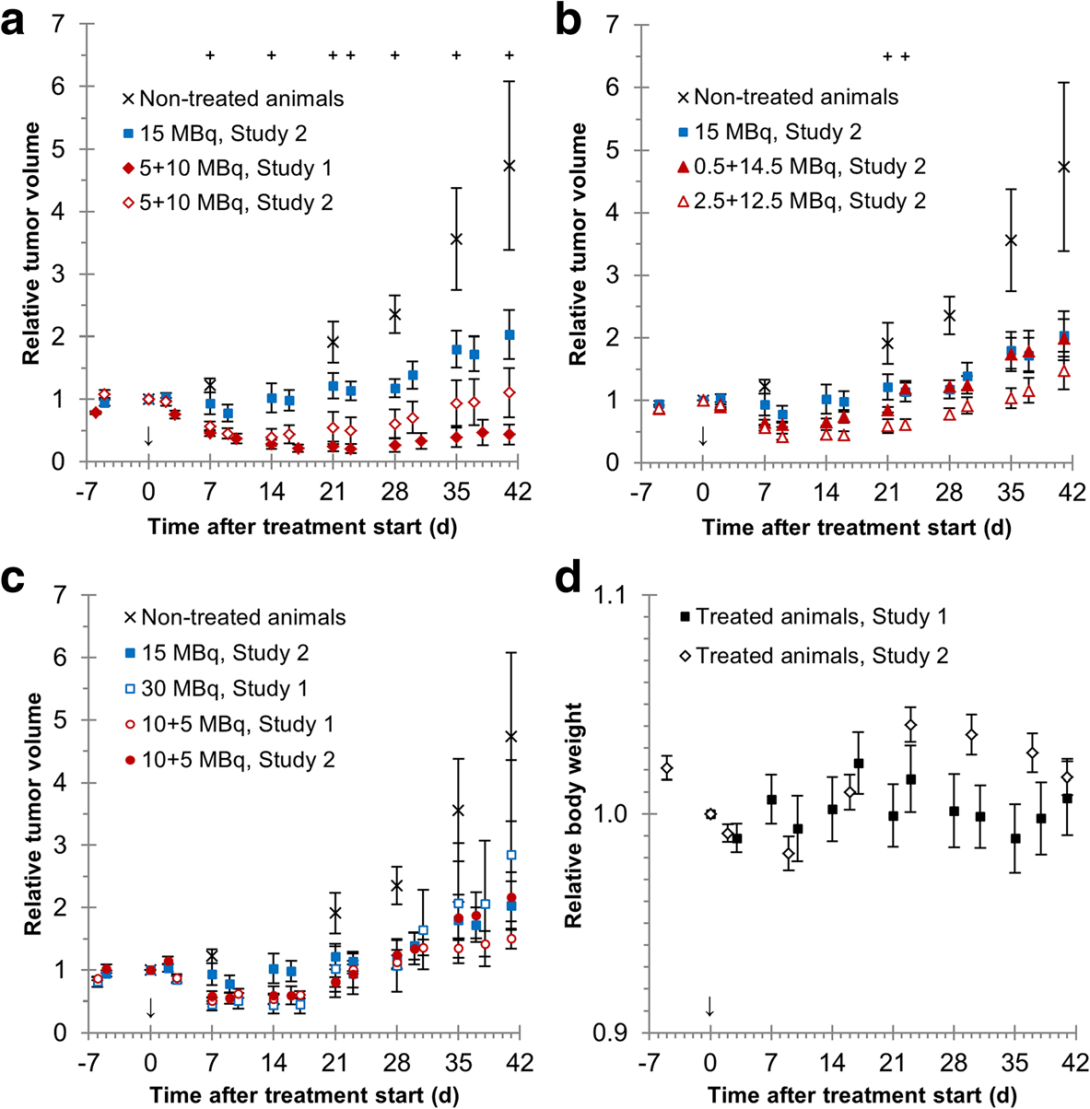
Anti-tumor effect of ^177^Lu-octreotate on GOT1-bearing nude mice. The relative tumor volume vs. time after last administration is shown for studies 1 and 2. **a** Evaluation of the anti-tumor effect of the priming treatment schedule used in the biodistribution and dosimetry study (5 + 10 MBq). **b** Results from groups receiving a smaller priming administration and larger subsequent administration than in the biodistribution and dosimetry study (0.5 + 14.5 and 2.5 + 12.5 MBq, respectively). **c** Anti-tumor effect in groups receiving a larger priming administration and smaller subsequent administration than in the biodistribution and dosimetry study (10 + 5 MBq) or a larger single administration (30 MBq). Measurements for non-treated animals and animals treated with 15 MBq ^177^Lu-octreotate are also shown in **a**–**c** for comparison. **d** Mean animal body weight in therapeutic studies 1 and 2 (note the scale of the *y* axis). The relative tumor volume reduction was statistically significant in the 2.5 + 12.5 MBq group and 5 + 10 MBq group (study 1 but not study 2) compared with the 15 MBq group (Student’s *t* test, *p* < 0.05), shown as the *plus symbol* at the top of the graph in **a** and **b**, respectively. *Symbols* represent mean relative tumor volumes. *Error bars* indicate SEM. *Black arrow* indicates start of treatment

The results from the therapeutic studies 1 and 2 are shown together, demonstrating the relative tumor volume vs. time after administration in animals receiving the treatment schedules used in the biodistribution and dosimetry study (5 + 10 and 15 MBq, Fig. 3a), a smaller priming administration and larger subsequent administration (0.5 + 12.5 and 2.5 + 12.5 MBq, Fig. 3b), and a larger priming administration and smaller subsequent administration or a larger single administration (10 + 5 and 30 MBq, Fig. 3c). The smallest relative tumor volumes were reached after 9–16 days in the groups receiving a priming administration, with the best therapeutic effects in the 2.5 + 12.5 MBq and 5 + 10 MBq groups. A statistically significant difference in minimum relative tumor volume was observed between these treatment groups and the 15 MBq group (*p* < 0.05). In the groups receiving a priming activity, the minimum relative tumor volumes were largest in the 0.5 + 14.5 MBq and 10 + 5 MBq groups but the mean values were generally smaller than if a single administration was given (15 and 30 MBq). No statistically significant adverse effects on animal weight were seen in either study 1 or study 2 (Fig. 3d, note the scale of the *y* axis). All measurement data on relative tumor volumes with statistical test results between all groups are available in Additional file 1: Table S1.

The tumor regrowth period, i.e., the time for the tumor to grow back to the tumor volume at the start of the treatment, was 21–28 days for the groups treated with 0.5 + 14.5, 2.5 + 12.5, 10 + 5 (studies 1 and 2), 15, and 30 MBq ^177^Lu-octreotete. However, the tumor regrowth time was longer, i.e., 35–41 days, for tumors treated with 5 + 10 MBq (studies 1 and 2).

### MRI examinations suggest heterogeneity of GOT1 xenografts

Tumors in all animals but one (15 MBq group, killed 72 h after injection, excluded from the study as described above) were well visualized in the MR images (Fig. 2).

**Fig. 4:**
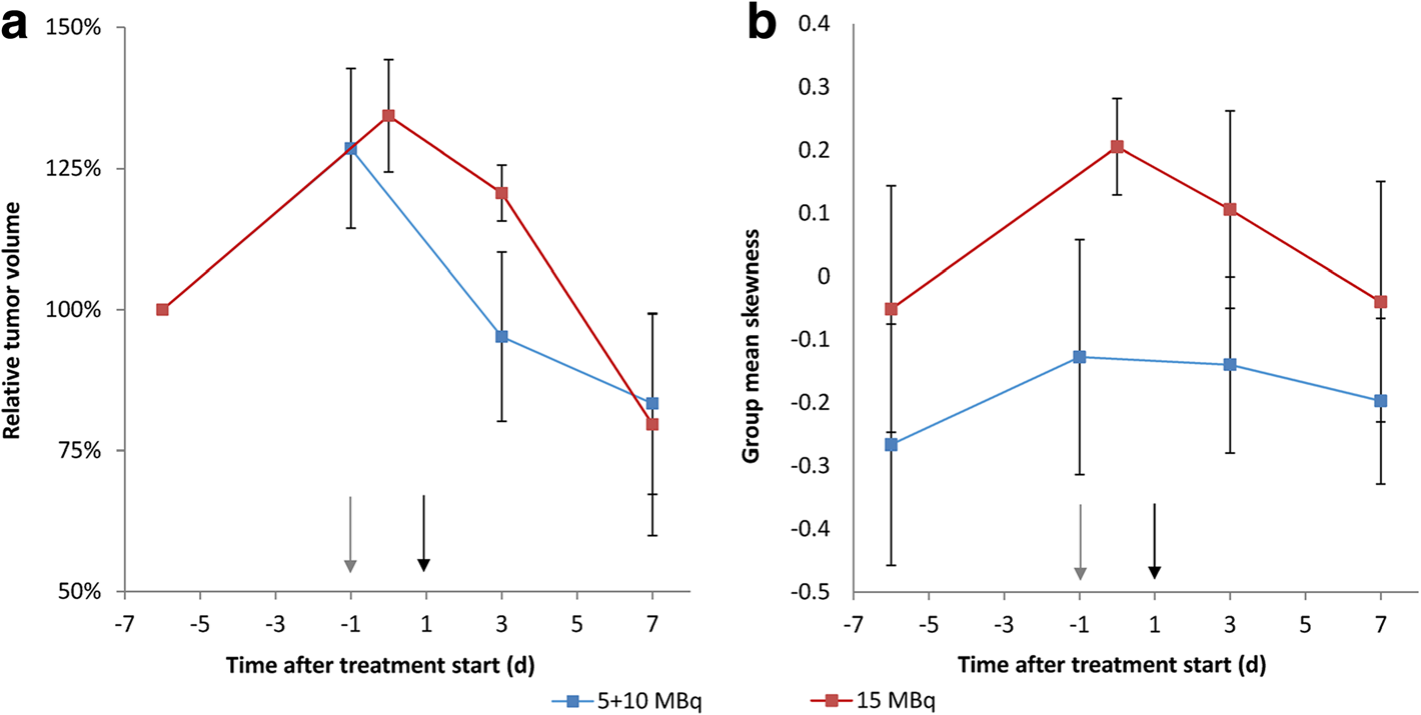
MRI of GOT1 xenograft in the biodistribution and dosimetry study. **a** Relative mean tumor volume for 5 + 10 and 15 MBq groups vs. time after treatment. The mean tumor volume in the 5 + 10 MBq group decreased by 5.7%/day after treatment, whereas the corresponding value was 5.1%/day in the 15 MBq group. **b** The mean skewness of the tumor voxel histograms is shown vs. time after administration. The general trend was a shift towards more positive skewness before treatment, with a reversed direction after treatment, where the negative shift is more pronounced in the 15 MBq group (*red line*). The *gray arrow* indicates the injection of 5 and 15 MBq and the *black arrow* indicates injection of 10 MBq. *Error bars* indicate SD

On average, tumor volumes determined by calipers were 20% smaller than those of the resected tumors. Corresponding MRI volumes were 1% larger than the resected tumors. The mean tumor volume regression rate after treatment was slightly higher in the priming group: −5.7 vs. −5.1%/day, but the difference was not statistically significant (*p* = 0.68) (Fig. 4a) nor were there statistically significant differences in the group mean tumor volumes after treatment (day 3: *p* = 0.22, day 7: *p* = 0.59, and day 3 + 7: *p* = 0.38).

The voxel intensity analysis revealed a trend common for both groups, where skewness increased before treatment, but decreased monotonically after treatment (Fig. 4b). The skewness in the 15 MBq group seemed to decrease at a higher rate than the 5 + 10 MBq group, but the difference was not statistically significant (*p* = 0.31).

### Enhanced pro-apoptotic and anti-proliferative transcriptional response in GOT1 tumors after priming administration

**Fig. 5:**
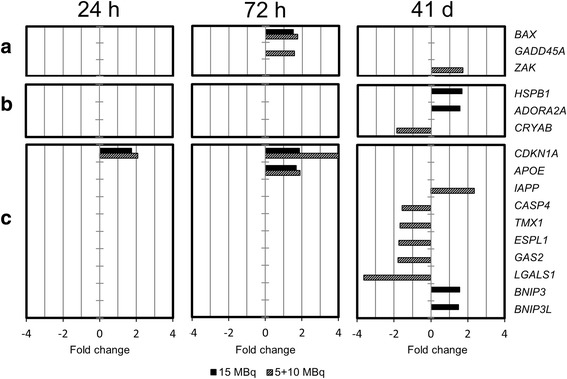
Expression of genes associated with cell death. Differential expression of genes associated with apoptotic cell death (**a** pro-apoptotic genes, **b** anti-apoptotic genes, **c** general apoptosis-related genes) in tumor samples from animals treated with 15 or 5 + 10 MBq ^177^Lu-octreotate, compared with untreated controls after 24 h, 72 h, and 41 days after the last injection. No genes related to non-apoptotic cell death were found in the analysis, and no genes associated with apoptotic cell death were found at 168 h after injection. Transcripts with changed expression ≥1.5-fold and an adjusted *p* < 0.01 were considered significantly regulated; association to biological process was performed using the GO database with a *p* value cutoff of <0.05

**Fig. 6:**
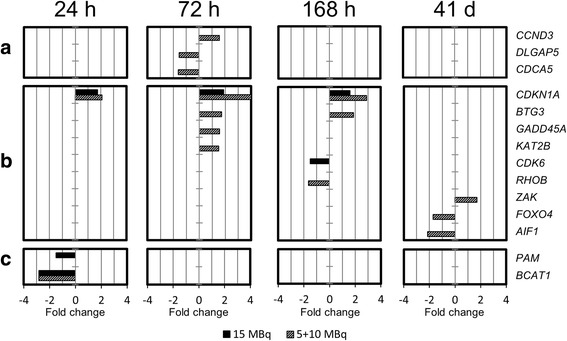
Expression of genes associated with cell cycle regulation. Differential expression of genes associated with cell cycle regulation (**a** pro-proliferative genes, **b** anti-proliferative genes, **c** general cell cycle regulation-related genes) in tumor samples from animals treated with 15 or 5 + 10 MBq ^177^Lu-octreotate, compared with untreated controls after 24 h, 72 h, 168 h, and 41 days after the last injection. Transcripts with changed expression ≥1.5-fold and an adjusted *p* < 0.01 were considered significantly regulated; association to biological process was performed using the GO database with a *p* value cutoff of <0.05

In total, 91, 380, 544, and 531 transcripts were significantly regulated in animals treated with 5 + 10 MBq ^177^Lu-octreotate at 24 h, 72 h, 168 h, and 71 days, respectively; 159, 214, 378, and 405 were the corresponding numbers in animals treated with 15 MBq ^177^Lu-octreotate. No significant regulation of *SSTR1–5* or the neuroendocrine markers chromogranin A (*CHGA*) and synaptophysin (*SYP*) was found in the global transcriptional analysis. Figures 5 and 6 show the differentially regulated genes (treated vs. untreated) associated with apoptotic cell death and cell cycle regulation in tumor samples from animals injected with 5 + 10 or 15 MBq ^177^Lu-octreotate 24 h, 72 h, 168 h, and 41 days after the last injection. The genes associated with apoptotic cell death were divided into pro-apoptotic, anti-apoptotic, and general apoptosis-related genes (Fig. 5a–c), according to GO-term association. A larger number of up-regulated pro-apoptotic genes were found in the 5 + 10 MBq group (*BAX*, *GADD45A*, and *ZAK*) compared to the 15 MBq group (*BAX*). One down-regulated gene (*CRYAB*) was found among the anti-apoptotic genes in the 5 + 10 MBq group, while two anti-apoptotic genes (*HSPB1* and *ADORA2A*) were significantly up-regulated in the 15 MBq group at 41 days after the last injection. No genes were significantly associated with apoptotic cell death at 168 h after injection, and genes associated with non-apoptotic cell death were not differentially regulated in any treatment group at any time point. In order to validate alterations in apoptosis-related genes after ^177^Lu-octreotate, we analyzed the expression of BAX protein in xenografted tumors by immunohistochemistry. The BAX protein was found to be strongly expressed in all tumors, with no difference in staining intensities between experimental groups.

Genes associated with cell cycle regulation were divided into pro-proliferative, anti-proliferative, and general cell cycle regulation-related genes (Fig. 6a–c). Three pro-proliferative genes were regulated in the 5 + 10 MBq group at 72 h after administration, one up- (*CCND3*) and two down-regulated (*DLGAP5* and *CDCA5*). A larger amount of anti-proliferative genes were regulated in the 5 + 10 MBq group (*CDKN1A*, *BTG3*, *GADD45A*, *KAT2B*, *CDK6*, *RHOB*, *ZAK*, *FOXO4*, and *AIF1*) compared to the 15 MBq group (*CDKN1A* and *CDK6*), and most genes were up-regulated. In order to validate the anti-proliferative effects after ^177^Lu-octreotate, we analyzed the percentage of Ki67-positive tumor cells in xenografted tumors by immunohistochemistry. We confirmed a reduction of Ki67-positive tumor cells in xenografts from treated animals compared to untreated controls (Table 1 (C)).

**Fig. 7:**
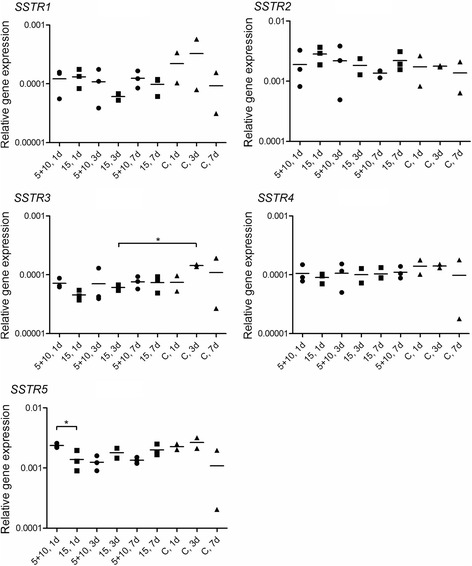
Expression of *SSTR1–5* in GOT1 tumors after ^177^Lu-octreotate exposure. Gene expression of *SSTR1–5* relative to *GAPDH* and *TUBB* analyzed by qRT-PCR in GOT1 tumors. Tumors were analyzed 1, 3, and 7 days after treatment with 5 + 10 MBq priming or 15 MBq ^177^Lu-octreotate as a single dose. Each *symbol* represents one tumor. *C* untreated control tumors. *Line* represents mean of triplicates. *p < 0.05

The gene expression of *SSTR1–5* was analyzed by qRT-PCR. Priming administration of 5 MBq followed by 10 MBq of ^177^Lu-octreotate 24 h later did not change the *SSTR1*–*4* gene expression in the tumors when compared with tumors that had received a single dose of 15 MBq ^177^Lu-octreotate or untreated tumors (Fig. 7). One day after treatment, *SSTR5* was found down-regulated in tumors treated with a single administration compared with priming administration. However, the *SSTR5* expression in tumors exposed to a single dose was not changed when compared with untreated tumors.

### Regressive changes in GOT1 xenografts after ^177^Lu-octreotate

**Fig. 8:**
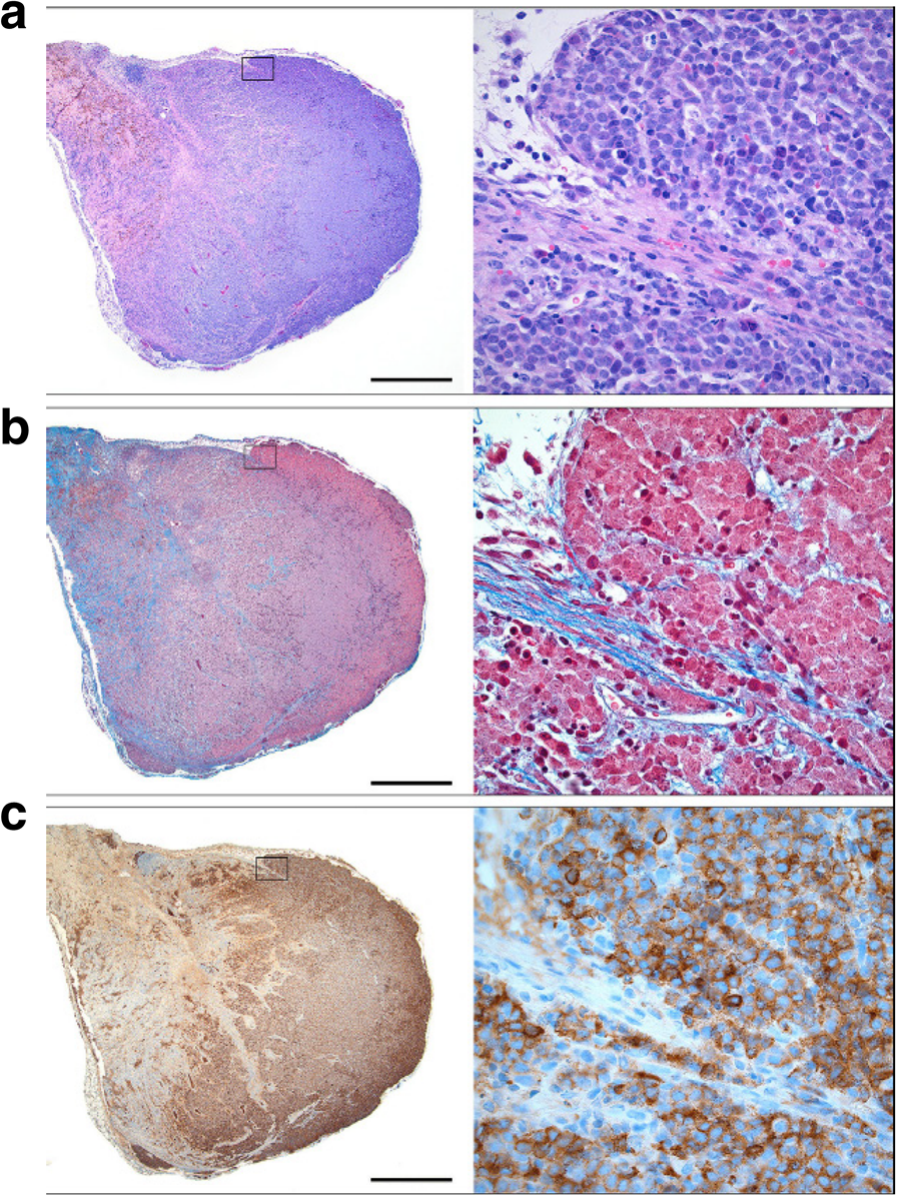
Morphology of GOT1 xenograft after ^177^Lu-octreotate exposure. This tumor was harvested from a mouse treated with a priming administration (2.5 MBq) followed by a subsequent dose (12.5 MBq) of ^177^Lu-octreotate 24 h later. Maximal reduction of tumor volume (67%) was noted 9 days after treatment. Thereafter, the tumor started to grow and was harvested on day 41 after the last injection. **a** The xenograft contained areas of fibrosis as well as areas of viable tumor cells (*left*). Viable tumor cells from the *boxed area* are shown at high magnification (*right*). Hematoxylin and eosin stain. **b** Tumor regression in xenografts is highlighted by staining for collagen (*blue*, Masson’s trichrome stain). **c** The NE differentiation of the xenograft was confirmed by positive staining for chromogranin A (*brown*). Viable tumor cells had a granular cytoplasmic staining as shown at high magnification (*right panel*). Scale bars equal 1 mm (*left panel*) and 50 μm (*right panel*)

Microscopic examination of xenografts in the biodistribution and dosimetry studies confirmed NE tumors with a morphology typical of GOT1. The percentage of tumor cells was in the 70–90% range. Examination of xenografts from the therapeutic studies showed heterogeneity of tumors, with viable NE tumor cells alternating with areas of fibrosis and inflammation, characteristic for tumor regression. Immunohistochemical staining for the NE markers chromogranin A and synaptophysin showed strong staining of the tumor cells (cf Fig. 8).

Histologic grading of tumor regression in the therapeutic study showed marked tumor regression (grades 1a and 1b) only in tumors treated with priming.

## Discussion

In the present study, we evaluated the possibility to increase the tumor-specific uptake of ^177^Lu-octreotate in GOT1 tumor-bearing BALB/c nude mice. A priming administration of ^177^Lu-octreotate was given 24 h before a subsequent administration to evaluate whether a better anti-tumor effect was obtained compared with that obtained after a single administration. We show that the mean absorbed dose to the tumor was higher after priming, and the anti-tumor effects were more pronounced in some of these groups as compared with a single administration. Differences in response after priming were also illustrated by gene expression analysis. Due to a lower mean absorbed dose to the kidneys, priming also resulted in an increased therapeutic window.

The activity concentration in the tumor was higher using a priming administration and increased until 72 h after the last injection. This increase could be explained by higher SSTR expression, since uptake can continue as long as ^177^Lu-octreotate is present in the blood. In several in vivo studies with ^177^Lu-octreotate treatment of NE tumors, rapid tumor shrinkage has been associated with a long retention time or even increasing tumor activity concentration over time [1, 13, 29]. This type of uptake and retention of ^177^Lu-octreotate was also found in the present study, with a significantly higher uptake after 72 h in the priming group (5 + 10 MBq) compared with the 15 MBq group. However, ^177^Lu-octreotate was probably degraded and released faster from the cells after 5 + 10 MBq than after 15 MBq administrations. The decrease in activity concentration in the tumor was nearly 60% between 72 and 168 h in the 5 + 10 MBq group compared with a 25% decrease for the 15 MBq group. Despite these results, the activity concentration (in %IA/g) was still higher in the 5 + 10 MBq group at 168 h.

In non-tumoral tissue, the mean activity concentration after 24 h was higher in the SSTR-expressing tissues after priming (5 + 10 MBq group), which indicates that SSTR could also be up-regulated in these tissues. These findings are not in accordance with previous results, which did not show any increased ^177^Lu-octreotate uptake in normal tissues [12]. An additional explanation to the higher activity concentrations found in tumoral and non-tumoral SSTR-expressing tissues might be that the priming administration influences vascular perfusion. Previous studies have shown an increase in perfused blood vessels in tumor tissue shortly after irradiation with X-rays (studied with Hoechst 33342 and DCE-MRI). This was followed by a decrease in perfusion at later times after irradiation, and a subsequent increase to control levels at 7 to 11 days after irradiation [30, 31]. This indicates that priming might increase perfusion in the tissue, resulting in an increased tumor uptake of the second injection of ^177^Lu-octreotate. Since the activity concentration was retained to a higher extent in the tumor tissue than in the kidneys and in the blood, the T/N ratios for the kidneys and blood were higher after priming, which is reflected by differences in mean absorbed dose. These findings indicate that the risk of renal and bone marrow toxicity would be lower or similar if priming was used, which would expand the therapeutic window; the activity concentration measured in the blood has been shown to closely mimic the activity concentration in the bone marrow [32]. Nevertheless, the mechanisms for increased ^177^Lu-octreotate uptake in tumor and non-tumor SSTR-expressing tissues still need to be identified.

To study the anti-tumor effect of priming, the treatment schedules used in the biodistribution and dosimetry study (5 + 10 and 15 MBq) were studied again in the therapeutic studies. However, since the 15 MBq treatment resulted in a lower mean absorbed dose to the tumors, a higher single administration activity (30 MBq) was also studied. Several other treatment schedules involving differently sized priming and subsequent administrations (all equaling a total of 15 MBq) were also studied in the therapeutic setting, in order to establish the optimal ratio between priming and subsequent administration amounts. The relative volumes from each group at each measurement time were tested using the Lilliefors test. The null hypothesis that the data come from a normally distributed population could not be rejected in any group at any time point (*p* value threshold <0.05). We therefore decided that the *t* test was valid for these comparisons. The best overall therapeutic effect was found for the 5 + 10 MBq groups. Lower and higher priming activities were not as effective even though a statistically significant difference was seen between the 15 MBq and 2.5 + 12.5 MBq groups when the minimum tumor volume was reached (days 16–23). To further optimize ^177^Lu-octreotate therapy, and increase the cure rate, the long-term anti-tumor effects of priming must be examined in more detail. Not surprisingly, the comparison between the relative tumor volumes in treated and non-treated animals showed that both types of treatment schedules (with or without a priming administration) are better than no treatment at all. However, statistically significant differences in mean relative tumor volume between treated and untreated animals were generally prolonged in time in animals receiving a priming treatment schedule, compared with the single administration groups. No statistically significant body weight loss was observed in either of the therapeutic studies. However, further studies on the difference in long-term adverse effects between different treatment schedules are needed, especially concerning adverse effects on, e.g., the kidneys.

The growth rate of GOT1 tumors differs much between animals within a transplantation batch, which results in a large variation in tumor sizes at a certain time point. Therefore, efforts were made to obtain a similar distribution of tumor sizes between the groups, since an inverse linear relationship between tumor size and ^177^Lu-octreotate concentration has been seen in another tumor type, the NCI-H69 small cell lung cancer [33], although no such data are found for GOT1. Furthermore, the tumor take is relatively low for GOT1, which also has a long tumor doubling time. Therefore, despite large efforts, variations in tumor size distribution between groups were inevitable, although no statistically significant difference in tumor volume was seen between any of the treatment groups before treatment start. This variation can be one reason why, e.g., the tumor regression in studies 1 and 2 for the 5 + 10 MBq group deviated.

In the present study, a total activity of 15 MBq (0.6-μg peptide) was chosen (a) to avoid complete tumor remission and (b) to be able to assess whether priming administration improves the therapeutic effect of ^177^Lu-octreotate. In a previous study on nude mice transplanted with GOT1 tumors, some of the mice that received 30 MBq ^177^Lu-octreotate (with a peptide amount of 1 μg) obtained complete tumor remission [1]. The moderate peptide amount was also chosen to avoid possible receptor saturation [13, 33]. Saturation was also avoided by giving the second administration no earlier than 24 h after the priming activity. During this time period, the ^177^Lu-octreotate in the priming administration would have had time to internalize and the receptors to recycle to the cell membrane [13, 34, 35]. In AR42J tumors transplanted in Lewis rats, internalization was fast (within 10 min) and recycling was completed within 24 h after administration of the somatostatin analog ([Tyr^3^, Thr^8^]-octreotide, TATE) [36].

Irradiation-induced SSTR up-regulation was not confirmed with either microarray or qRT-PCR analysis after either priming or single administration. This was unexpected, since we have previously demonstrated radiation-induced up-regulation in vitro (by qRT-PCR), and an in vivo study demonstrated increased concentration of radiolabeled octreotide after priming but without a direct proof of *SSTR* up-regulation [10–13]. However, one probable reason why no up-regulation of SSTR was found in the present study is that the analyses were made at too late time points (24 h, 72 h, 168 h, and 41 day after the last injection). Down-regulation of *SSTR2* after a single injection of octreotide in AR42J-bearing CB-17 SCID mice was shown already after 0.5 h and with a complete cell surface recovery of *SSTR2* after 16–24 h [37]. In the same study, differences between a continuous release with mini-osmotic pumps of octreotide (daily) and discontinuous administrations of octreotide (given twice daily) were found, with increasing and decreasing tumor octreotide uptake, respectively. The kinetics of SSTR expression are clearly time dependent, and further studies are needed to clarify the mechanisms by which priming improves the uptake of ^177^Lu-octreotate in tumor tissue, as well as the dose-response relation between administered activity and SSTR expression. We have previously reported an increased tumor uptake of ^111^In-octreotide following 7.5 MBq ^177^Lu-octreotate in GOT1 xenografts, but not following 30 MBq ^177^Lu-octreotate [12, 13]. Thus, the 15 MBq single administration may be too high to result in detectable *SSTR* up-regulation. Furthermore, the 5 + 10 MBq group presents a convoluted view of the gene expression response to both the 5 MBq priming administration and to the subsequent 10 MBq administration.

Caliper measurements of tumor volume are prone to measurement errors stemming from, e.g., skin thickness, operator bias, and differences in tumor shape and location. These errors may conceal small volume changes associated with early or small treatment effects. MRI-based tumor volume determination has been shown to better reflect the true tumor volume, especially for small tumors, which should effectively increase the sensitivity of treatment assessments [22], a result confirmed by the present study. Nevertheless, the MRI method could not separate the effects in the 5 + 10 MBq group from the 15 MBq group in the biodistribution and dosimetry study, although confounding factors, such as differences in pre-treatment growth kinetics and tumor size, were minimal.

MRI allows non-invasive monitoring of local intra-tumor treatment response due to the effects on tissue relaxation times (T1 and T2) and hence image contrast. Increased T1 and decreased T2 values have been found in tumors responding to chemotherapy [38, 39] which would result in a more positively skewed voxel intensity distribution for T2W images. However, changes in tumor size would also affect the voxel intensity distribution if the relative volume reduction of hypo- and hyper-intense tissue regions was not equal. In this study, tumor volume decreased similarly in both treatment groups, but skewness plateaued in the priming group, which indicates a greater relative reduction of hyper-intense (viable) tumor tissue. Neither volume nor skewness could separate the groups on a statistically significant level, but the reduced volume with maintained skewness supported our assumption of increased treatment effect size after priming.

To the best of our knowledge, there are no reports on the transcriptional responses in tumor tissue after ^177^Lu-octreotate therapy in vivo. In the present study, tumor samples from animals receiving 5 + 10 or 15 MBq were analyzed with respect to global transcriptional response. These groups were selected to compare the transcriptional effects in tumors from the priming group with the smallest minimum relative tumor volume (5 + 10 MBq study 1; mean relative volume 0.21 at 23 days) with those in tumors from the single administration group with the largest minimum relative tumor volume (15 MBq study 2; mean relative volume of 0.78 at 9 days). When setting the statistical thresholds for the transcriptional analysis, the biological interpretation needs to be considered in relation to the sensitivity of the analysis. The fold-change threshold of 1.5 was chosen as a compromise between a complete view of the transcriptional response and omitting responses that are not strong enough to be reproduced or may be the result of statistical noise. Our experience is that a fold change of 1.5 can be used with an acceptable sensitivity (especially in combination with the FDR-adjusted *p* value threshold of <0.01) and may also yield an understanding of potential downstream effects which are biologically relevant compared with the normal baseline gene expression [40, 41].

Results from the global transcriptional studies indicated that apoptotic cell death and cell cycle regulation are affected during the first days after ^177^Lu-octreotate treatment. At 72 h after injection, *BAX* was up-regulated in both treatment groups and *GADD45A* was up-regulated in the tumors from animals receiving a priming administration. Both *BAX* and *GADD45A* are regulated by *p53*, and are induced upon DNA damage to promote apoptosis [42]. *GADD45A* has also been suggested to act as a backup for *CDKN1A* (also known as *p21*), which is involved in p53-regulated DNA damage repair, cell cycle arrest, and apoptosis [42, 43]. That both of these genes were up-regulated after priming treatment and only to a lower (*CDKN1A*) or no (*GADD45A*) degree suggests a strong involvement of the p53 pathway in the cellular tumor response to a priming treatment with ^177^Lu-octreotate. At study end (41 days after last injection), a response in apoptosis-inhibiting processes was seen. In the 15 MBq group, two genes (*HSPB1* and *ADORA2A*) were up-regulated, while one gene (*CRYAB*) was down-regulated in the 5 + 10 MBq group. *HSPB1*, encoding a small heat shock protein, has been shown to reduce activity of the pro-apoptotic JNK signaling pathway (which acts via p53), promoting survival in cells under stress. Several of the other regulated apoptosis-related genes (*APOE*, *BAX*, *BNIP3*, *BNIP3L*, *IAPP*, and *LGALS1*) have also been shown to be associated with the JNK signaling pathway [44–48]. *CRYAB* (also known as *HSPB5*), encoding a heat shock protein similar to HSPB1, has previously been shown to be able to inhibit both the “intrinsic” and “extrinsic” apoptotic pathways [49]. These results, in combination with the up-regulation (after priming treatment) of *ZAK* (also known as *MLK7*), encoding a MAP3 kinase that has been shown to activate *JNK* [50], may explain the differences in tumor regrowth between groups receiving priming and single administration. Non-apoptotic-related cell death effects were not seen in the current data, which indicates that apoptosis was the main process involved in the reduction of tumor volume.

## Conclusions

In summary, we observed an almost twofold higher mean absorbed dose to tumor tissue when using priming administration compared with a single administration schedule. The mean absorbed dose to the dose-limiting organs—i.e., the kidneys and bone marrow—were lower or similar; therefore, we conclude that priming increases the anti-tumor effect as well as expands the therapeutic window of ^177^Lu-octreotate treatment.

Transcriptional analysis revealed activation of anti-apoptotic genes in tumors in the 15 MBq treatment group but pro-apoptotic activity in tumors from animals receiving the 5 + 10 MBq treatment schedule. These findings demonstrate that priming does not only influence the intensity of tumor response but also its biological quality with regard to cell survival. Several of the genes regulated after 41 days have previously been linked with JNK signaling, which may indicate that potential biomarkers for tumor regrowth may be found in the JNK signaling pathway.

These results of the present study might have clinical implications, and priming administration is an interesting optimization strategy for ^177^Lu-octreotate therapy of neuroendocrine tumors. Further studies should be performed to determine the mechanisms responsible for increased anti-tumor effects and increased therapeutic window obtained after priming administration.

## Additional file

Additional file 1:
**Table S1.** Anti-tumor effect of ^177^Lu-octreotate on GOT1-bearing nude mice. (DOCX 21 kb)

## Abbreviations

%IA/g: Percent of injected activity per gram tissue
^177^Lu-octreotate: ^177^Lu-[DOTA^0^, Tyr^3^]-octreotate
GO: Gene Ontology
MRI: Magnetic resonance imaging
NE: Neuroendocrine
qRT-PCR: Quantitative reverse transcription-polymerase chain reaction
SD: Standard deviation
SEM: Standard error of the mean
SI-NET: Small intestine-neuroendocrine tumor
SSTR: Somatostatin receptor
T/N: Tumor-to-normal tissue activity concentration ratio
