# Supplementary material for: Priming increases the anti-tumor effect and therapeutic window of 177Lu-octreotate in nude mice bearing human small intestine neuroendocrine tumor GOT1
Source: EJNMMI Res. 2017 Jan 5;7:6. doi: 10.1186/s13550-016-0247-y (PMC5241264; doi:10.1186/s13550-016-0247-y)
Supplement: Additional file 1: — Table S1. Anti-tumor effect of 177Lu-octreotate on GOT1-bearing nude mice. (DOCX 21 kb) [file 13550_2016_247_MOESM1_ESM.docx]

| **Additional file 1: Table S1 Anti-tumor effect of ^177^Lu-octreotate on GOT1-bearing nude mice** | | | | | | | | | | | | | | | | | | |
| --- | --- | --- | --- | --- | --- | --- | --- | --- | --- | --- | --- | --- | --- | --- | --- | --- | --- | --- |
|  | **Relative tumor volume** | | | | | | | | | | | | | | | | | |
| **Therapeutic study 1** | **-6 d** | **0 d** | **3 d** | **7 d** | **10 d** | | **14 d** | **17 d** | | **21 d** | **23 d** | **28 d** | | **31 d** | **35 d** | | **38 d** | **41 d** |
| 5+10 MBq | 0.78 (0.02) | 1 (0) | 0.75 (0.06) | 0.45 (0.05)^a,d,e^ | 0.37 (0.08) | | 0.28 (0.07)^a,d^ | 0.21 (0.06) | | 0.24 (0.08)^a,d,e^ | 0.21 (0.07)^a,b,d^ | 0.26 (0.11)^a,b,d,e^ | | 0.33 (0.13) | 0.38 (0.16)^a,b,d^ | | 0.47 (0.20) | 0.43 (0.16)^a,b,c,d^ |
| 10+5 MBq | 0.86 (0.06) | 1 (0) | 0.87 (0.12) | 0.51 (0.19)^f^ | 0.63 (0.25) | | 0.54 (0.25) | 0.60 (0.25) | | 0.81 (0.24) | 1.0  (0.3) | 1.1  (0.4) | | 1.4 (0.5) | 1.3  (0.5) | | 1.4 (0.5) | 1.5  (0.5) |
| 30 MBq | 0.82 (0.08) | 1 (0) | 0.84 (0.06) | 0.46 (0.10)^g^ | 0.51 (0.12) | | 0.45 (0.15) | 0.46 (0.15) | | 1.0  (0.4) | 0.94 (0.33) | 1.1  (0.4) | | 1.6 (0.6) | 2.1  (1.0) | | 2.1 (1.0) | 2.9  (1.5) |
| **Therapeutic study 2** | **-5 d** | **0 d** | **2 d** | **7 d** | **9 d** | | **14 d** | **16 d** | | **21 d** | **23 d** | **28 d** | | **30 d** | **35 d** | | **37 d** | **41 d** |
| 0.5+14.5 MBq | 0.87 (0.07) | 1 (0) | 0.90 (0.04) | 0.64 (0.05)^a,j^ | 0.61 (0.04) | | 0.65 (0.06)^a^ | 0.74 (0.10) | | 0.85 (0.05)^a,j^ | 1.2 (0.1)^a,h,i^ | 1.2  (0.1)^a,h,j^ | | 1.2 (0.1) | 1.7  (0.3)^a^ | | 1.8 (0.3) | 2.0  (0.3)^a^ |
| 2.5+12.5 MBq | 0.87 (0.05) | 1 (0) | 0.94 (0.04) | 0.57  (0.05) | 0.41 (0.05) | | 0.46 (0.07) | 0.45 (0.06) | | 0.59 (0.11) | 0.61 (0.08)^b,h^ | 0.78 (0.09)^b,h^ | | 0.92 (0.13) | 1.0  (0.2)^b^ | | 1.2 (0.2) | 1.5  (0.3)^b^ |
| 5+10 MBq | 1.1 (0.07) | 1 (0) | 0.96 (0.06) | 0.56 (0.08)^l^ | 0.45 (0.08) | | 0.39 (0.14) | 0.44 (0.14) | | 0.54 (0.26)^k,l^ | 0.50 (0.21)^i,k^ | 0.60  (0.23)^l^ | | 0.70 (0.25) | 0.94 (0.36) | | 0.96 (0.37) | 1.1  (0.40) |
| 10+5 MBq | 1.0  (0.1) | 1 (0) | 1.1  (0.1) | 0.58 (0.09)^m^ | 0.55 (0.10) | | 0.60 (0.17) | 0.60 (0.17) | | 0.82 (0.25) | 0.94 (0.24) | 1.2  (0.5) | | 1.3 (0.4) | 1.8  (0.6) | | 1.9 (0.5) | 2.2  (0.6)^c^ |
| 15 MBq | 0.94 (0.05) | 1 (0) | 1.0  (0.1) | 0.93 (0.18)^d^ | 0.78 (0.14) | | 1.0  (0.2)^d^ | 0.98 (0.16) | | 1.2 (0.2)^d,k^ | 1.1 (0.2)^d,k^ | 1.2  (0.1)^d,n^ | | 1.4 (0.2) | 1.8  (0.3)^d^ | | 1.7 (0.3) | 2.0  (0.4)^d^ |
| **Controls** | **0 d** | **7 d** | | | | **21 d** | | | **28 d** | | | | **35 d** | | | **41 d** | | |
|  | 1 (0) | 1.2 (0.1)^e,f,g,j,l,m^ | | | | 1.9 (0.3)^e,j,l^ | | | 2.4 (0.3)^e,j,l,n^ | | | | 3.6 (0.8) | | | 4.7 (1.3) | | |
| The relative tumor volume at different time points after the last administration of ^177^Lu-octreotate in the different treatment groups. Values are given as mean (SEM). Statistically significant difference between groups is indicated by: ^a^5+10 MBq study 1 *vs.* 0.5+14.5 MBq, ^b^5+10 MBq study 1 *vs.* 2.5+12.5 MBq, ^c^5+10 MBq study 1 *vs.* 10+5 MBq study 2, ^d^5+10 MBq study 1 *vs.* 15 MBq, ^e^5+10 MBq study 1 *vs.* Control, ^f^10+5 MBq study 1 *vs.* Control, ^g^30 MBq *vs.* Control, ^h^0.5+14.5 MBq *vs.* 2.5+12.5 MBq, ^i^0.5+14.5 MBq *vs.* 5+10 MBq study 2, ^j^0.5+14.5 MBq *vs.* Control, ^k^5+10 MBq study 2 *vs.* 15 MBq, ^l^5+10 MBq study 2 *vs.* Control, ^m^10+5 MBq study 2 *vs.* Control, ^n^15 MBq *vs.* Control. | | | | | | | | | | | | | | | | | | |
